# SOCIAL ENGAGEMENT MEDIATES THE ASSOCIATION BETWEEN ROLE OVERLOAD AND NIGHTTIME AWAKENING OF DEMENTIA CAREGIVERS

**DOI:** 10.1093/geroni/igz038.2495

**Published:** 2019-11-08

**Authors:** Jiaming Liang, Maria P Aranda, Donald Lloyd

**Affiliations:** University of Southern California, Los Angeles, California, United States

## Abstract

Caregivers tend to report role overload and nighttime awakening given the burden of caregiving. Previous research has not explored the association between role overload and nighttime awakening among dementia caregivers. Social engagement has been found to be associated with physical and psychological health outcomes of caregivers. Thus, the present study aims to examine whether role overload is associated with nighttime awakening of dementia caregivers and whether social engagement will mediate the association. We conducted a cross-sectional study by using the 2015 National Health and Aging Trends Study (NHATS) and National Study of Caregiving (NSOC). Six-hundred-and-sixty-nine dementia caregivers were included in the analysis. Relevant factors were controlled as covariates including age, gender, race/ethnicity, etc. Nearly 45% of participants reported suffering from nighttime awakening at “some” or “more” nights within one month, among which half of them reported “almost” or “every” night. Role overload was found associated with caregivers’ nighttime awakening (β=.135, 95%CI: .094 to.176, p<.001). The mediated model shows 7.4% of association between role overload and nighttime awakening could be explained by the mediation of social engagement (β= .010, 95%CI: 0.99x10-2 to 1.01x10-2, p<.05). The study suggests that dementia caregivers with high levels of role overload tend to experience nighttime awakening more than those who reported low role overload. A modest mediated effect indicates that role overload of dementia caregivers can predict their nighttime awakening partly through decreasing their social engagement. Other factors and longitudinal models shall be discussed to further explore the theoretical mechanisms of caregiver stress.

